# CsbD, a Novel Group B Streptococcal Stress Response Factor That Contributes to Bacterial Resistance against Environmental Bile Salts

**DOI:** 10.1128/jb.00448-22

**Published:** 2023-05-17

**Authors:** Lu Jia, Chen Yuan, Fei Pan, Xiaorui Zhou, Hongjie Fan, Zhe Ma

**Affiliations:** a Ministry of Agriculture Key Laboratory of Animal Bacteriology, International Joint Laboratory of Animal Health and Food Safety, and College of Veterinary Medicine, Nanjing Agricultural University, Nanjing, Jiangsu, China; b Jiangsu Co-innovation Center for Prevention and Control of Important Animal Infectious Diseases and Zoonoses, Yangzhou, China; University of Chicago

**Keywords:** Group B *Streptococcus*, bile salt resistance, *csbD*

## Abstract

Group B Streptococcus (GBS) can cause many serious infections and result in severe symptoms depending on the infected organs. To survive and initiate infection from the gastrointestinal tract, GBS must resist physiochemical factors, such as bile salts, a potent antibacterial compound in the intestine. We found that GBS isolated from diverse sources all possess the capability to defend bile salts and permit survival. By constructing the GBS A909 transposon mutant library (A909_Tn_), we identified several candidate genes that might participate in the bile salt resistance of GBS. The *rodA* and *csbD* genes were validated as relevant to bile salt resistance. The *rodA* gene was anticipated to be related to peptidoglycan synthesis and influence the bile salt resistance of GBS by cell wall construction. Notably, we found that the *csbD* gene worked as a bile salt resistance response factor and influenced several ABC transporter genes, specifically at the later growth period of GBS under bile salt stress. We further detected the marked intracellular bile salt accumulation in Δ*csbD* by hydrophilic interaction chromatography-liquid chromatography/mass spectrometry (HILIC-LC/MS). Collectively, we showed a novel GBS stress response factor, *csbD*, contributes to bacterial survival in bile salts by sensing bile salt stress and subsequently induces transcription of transporter genes to excrete bile salts.

**IMPORTANCE** GBS, a conditional pathogenetic colonizer of the human intestinal flora, can cause severe infectious diseases in immunocompromised patients. Therefore, it is critical to understand the factors that contribute to the resistance to bile salts, which are abundant in the intestine but harmful to bacteria. We identified *rodA* and *csbD* genes involved in bile salt resistance using a transposon insertion site sequencing (TIS-seq) based screen. The *rodA* gene products might be involved in peptidoglycan synthesis as important contributors to stress resistance including bile salts. However, the *csbD* gene conferred bile salt resistance by promoting transporter genes transcription at the later growth period of GBS in response to bile salts. These findings developed a better understanding of the stress response factor *csbD* on the bile salt resistance of GBS.

## INTRODUCTION

Group B Streptococcus (GBS; Streptococcus agalactiae) is a commensal Gram-positive bacterium found in the gastrointestinal and genitourinary tracts of healthy humans and is also a potential pathogen that causes well-recognized infections in neonates and pregnant women (1). In recent decades, GBS has drawn attention because of the increasing incidence of its infection in nonpregnant adults (2–4), especially in the elderly and those with chronic medical conditions. In these sensitive populations, the GBS infection could be life-threatening and require hospitalization and intensive care unit management (5, 6). The increasing resistance to erythromycin and clindamycin of GBS, as well as high-level resistance to gentamicin and vancomycin, has been reported worldwide (7–9); thus, GBS has been considered a public health threat due to the emergence of drug-resistant isolates (10).

It is generally accepted that maternal colonization is the main cause of early onset GBS disease (11–13). The most frequently colonized site is the intestine, which is assumed to be the natural reservoir of GBS and the likely source of vaginal and oral colonization (14–16). In healthy adults, GBS predominantly colonizes in the colon yet may occasionally reside in the small intestine as well. For the development of disease caused by GBS spreading from the intestine, the bacterium has to colonize in the colon and potentially the small intestine (17). During intestinal colonization, the GBS is exposed to a stressful environment with diverse antibacterial substances, particularly bile salts. Bile is not only a kind of digestive secretion that mainly participates in the digestion and absorption of lipids, but it is also harmful to some bacteria that colonized the intestine as well. The bile salts may lead to bacterial death by DNA damage, protein misfolding, and membrane integrity disruption due to oxidative stress (18, 19). Although a majority of bile (95%) is recycled back into the small intestine by enterohepatic circulation, there is still 0.3 to 0.6 g of bile salts lost through feces per day, which is also the major contribution of colon bile salts (18). Therefore, to survive in the stressful environment of bile salts, bacteria have evolved a series of protective mechanisms to adapt to the bile salt-rich environment of the digestive tract. The major bile salt tolerance strategies of Listeria monocytogenes include (i) the outer membrane acts as a barrier to the influx of bile salts; (ii) the bile salt efflux contributes to maintaining ion balance; and (iii) bile salt hydrolase (BSH) hydrolyzes taurine-conjugated bile acids into free bile acid, which can be further metabolized by other gut bacteria (20, 21). Although the tolerance of high concentrations of whole bile in GBS has been documented, there is a significant gap in knowledge regarding how the GBS resistant to bile salts colonizes the gastrointestinal tract and causes infection (22).

Here, we found that GBS isolated from different sources possesses bile salt resistance capability generally. With the transposon insertion site sequencing (TIS-seq), we identified several candidate genes that contributed to the fitness of GBS under bile salt stress. The *rodA* gene involved in peptidoglycan synthesis was validated as essential for GBS survival under environmental stress, including but not limited to bile salts. The *csbD* gene was identified as relevant to bile salt resistance at the later growth period of GBS in response to bile salts. By transcriptomics and hydrophilic interaction chromatography-liquid chromatography/mass spectrometry (HILIC-LC/MS) analysis, we anticipated that the bile salt resistance capability of GBS conferred by the *csbD* gene was related to transporter genes that work as bile salt efflux factors. This study deepened our understanding of the bile salt resistance mechanism of the novel GBS stress response factor *csbD* by influencing transporter genes that might promote bile salt excretion during the later growth period of GBS in a bile salt stress environment.

## RESULTS

### Identification of GBS bile salt resistance genes with transposon insertion site sequencing (TIS-seq) screen.

**FIG 1:**
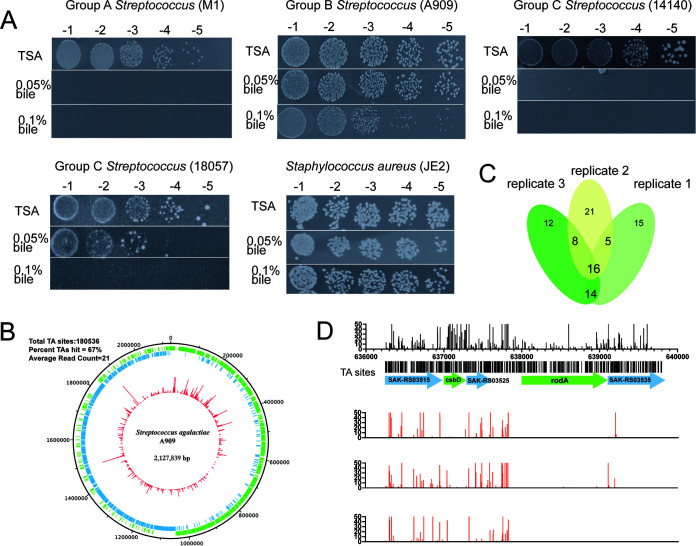
Identification of GBS bile salt resistance genes with TIS-seq screen. (A) The colonies of an isolate of GAS (M1), GBS (A909), GCS (14140 and 18057), and the *S.aureus* control (JE 2) at each serial dilution on TSA plates with or without 0.05%, 0.1% ox bile extract. (B) Circular representation of GBS Tn mutant library. Red rings show unique transposon insertion counts at each TA dinucleotide site. The green and blue rings indicate coding region (CDS) on different DNA strands. (C) The Venn diagram of the number of bile resistance genes screening outputs (top 50) from three biological replicates. (D) Independent insertions and their fitness effects in the genomic region from gene SAK_RS03515 to SAK_RS03535 in input Tn library and three biological replicates outputs are plotted.

The bile salt resistance capability of GBS in the gastrointestinal tract is essential for the establishment of infection and persistent colonization (17). To compare the bile salt resistance of GBS with other streptococcal species, 24 strains including group A Streptococcus (GAS), GBS, and group C Streptococcus (GCS) were serial diluted and spotted on tryptic soy agar (TSA) plates with 0.05 mg/mL or 0.1 mg/mL bile salts (Fig. S1A in the supplemental material). The representative colonies density of GAS (M1), GBS (A909), GCS (14140 and 18057), and the *S.aureus* control (JE 2) on TSA plates with 0.05 mg/mL or 0.1 mg/mL ox bile extract is shown in Fig. 1A. The number of GBS strain dilutions was similar among 0.05 mg/mL, 0.1 mg/mL bile salts, and control plain TSA plates, indicating that the resistance capability of bile salt was common in GBS strains from different sources. All of the GBS strains showed significantly higher bile salt resistance on TSA plates containing 0.05 mg/mL or 0.1 mg/mL bile salts compared to GAS and GCS. None of the GAS strains could survive even 0.05 mg/mL bile salts. Although some of the GCS strains (e.g., 18057) were partially resistant to 0.05 mg/mL bile salts, they were completely suppressed by 0.1 mg/mL bile salts. These data suggested that the bile salt resistance capability of GBS was not universal in all streptococcal species but somehow specific in GBS. As a known bile salt resistance Gram-positive coccus, the Staphylococcus aureus was used as the positive control.

A high-density TIS-seq-based strategy was used to identify loci that might contribute to bile salt resistance for GBS (23). We constructed the A909_Tn_ with *Himar1* transposon that was inserted in the TA dinucleotides site of the genome. In the A909_Tn_, we found that the insertion sites of the transposon covered 67% of the total 180,536 TA sites on the genome (Fig. 1B). Despite that there are some hot spots of the transposon insertion sites, 390 genes (11% of the total genes in GBS) were identified as essential, which was similar to a recent report (317 core essential genes in GBS genome in THY growth conditions) (24). In addition, 3,099 nonessential genes and 292 domain essential genes were identified as well (Fig. S1B). Therefore, the great quality of the library was acceptable for further screening.

We compared the relative frequencies of transposon insertions at each locus when the library grew in the absence versus the presence of 1-mg/mL bile salt (1%) Todd-Hewitt broth (THB) plate. The higher relative frequencies of transposon insertions at each locus indicated the gene contributed less to bile salt resistance. After three biological replicates of screening, 43 genes were identified as GBS bile salt resistance relevant genes by overlapping the top 50 genes of each two replicates (Fig. 1C). Most genes were categorized into cell wall synthesis function that is important for environmental stress resistance as the first physical barrier, such as *rodA* and *lta* (Table 1) (25, 26). In addition, bacteria tend to extensively use various carbon sources in a stressful environment to meet their survival needs (27), so the genes involved in glycolipid metabolisms such as diacyl glycerol kinase (DGK) and phosphatase PAP2 family protein were also identified in our screen (28). The rest of identified genes were categorized into ribosomal structure and biogenesis, enzymes involved in energy conversion reactions, as well as transport proteins. Finally, we focused on two candidate genes putatively involved in bile salt resistance based on the results of the current study (Table 1): *rodA*, coding the ubiquitous shape, elongation, division, and sporulation (SEDS) family proteins. It works in peptidoglycan synthesis, which is vital for cell wall integrity and responsible for bacterial survival under environmental stress, presumably participating in bile salt resistance (29). The other gene is *csbD*, encoding general stress response protein, and the relationship between its function and stress response to bile salts has not been investigated, especially in Streptococcus.

**TABLE 1:** The bile resistance candidate genes identified by TIS

Gene locus	Gene name	Log 2 (FC)			−Log 10 (*P*)		
		Replicate 1	Replicate 2	Replicate 3	Replicate 1	Replicate 2	Replicate 3
Cell wall synthesis							
SAK_RS03530	RodA cell cycle protein	−11.70	−4.88	−10.76	23.96	22.57	23.99
SAK_RS07115	LTA synthase family protein	−11.09	−12.95	−9.58	5.79	6.43	5.21
SAK_RS09125	Teichoic acid d-Ala incorporation-associated protein DltX	−12.34	−12.63	−10.83	6.33	6.52	6.33
SAK_RS04450	Penicillin-binding protein PBP2B	−12.12	−9.81	−12.20	46.93	49.65	47.10
SAK_RS02920	DivIVA domain-containing protein	−10.66	−9.35	−10.73	5.56	5.68	5.68
SAK_RS08680	Phosphatase PAP2 family protein	−11.62	−9.73	−12.70	19.57	19.73	19.86
SAK_RS04235	Cell wall metabolism sensor histidine kinase VicK	−10.10	−11.38	−12.17	26.32	27.88	26.91
SAK_RS04660	Peptidylprolyl isomerase PrsA	−12.85	−9.96	−10.11	21.99	22.74	22.12
SAK_RS03545	Septation ring formation regulator EzrA	−10.20	−11.46	−11.25	25.98	27.72	25.97
SAK_RS03585	LCP family protein	−11.35	−7.15	−13.01	36.75	35.93	36.95
SAK_RS07140	LysM peptidoglycan-binding domain-containing protein	−10.77	−11.04	−10.84	7.31	7.98	7.37
Stress response							
SAK_RS03520	CsbD family protein	−11.64	−10.33	−11.72	4.20	3.96	4.06
SAK_RS04565	Superoxide dismutase SodA	−11.57	−11.86	−11.65	13.91	14.14	13.96
SAK_RS08590	Asp23/Gls24 family envelope stress response protein	−11.84	−12.12	−11.91	11.63	11.87	11.67
Glycolipid metabolism							
SAK_RS07690	DGK	−12.06	−10.76	3.66	13.62	3.66	13.64
SAK_RS09050	Ribulose-phosphate 3-epimerase	−11.20	−11.48	17.24	16.50	17.24	16.31
SAK_RS07290	Glycosyltransferase family 2 protein	−10.74	−11.03	22.40	20.52	22.40	20.71
SAK_RS04180	Glycosyltransferase	−10.21	−10.77	26.98	27.28	26.98	27.61
SAK_RS04445	Phosphoglycerate mutase	−10.74	−10.02	1.53	1.42	1.53	1.44
Others							
SAK_RS07105	Type I 3-dehydroquinate dehydratase	−10.74	−11.01	16.65	16.00	16.65	16.06
SAK_RS09045	Thiamine diphosphokinase	−6.08	−11.22	15.80	14.22	15.80	14.86
SAK_RS04655	*O*-methyltransferase	−11.46	−8.41	16.96	16.95	16.96	17.03
SAK_RS03535	HAD-IA family hydrolase	−11.37	−11.65	−11.45	10.02	10.34	10.24
SAK_RS00420	Acyltransferase	−11.32	−10.00	−8.08	39.77	44.02	38.63
SAK_RS10580	Uridine-5-carboxymethylaminomethyl synthesis enzyme MnmG	−11.54	−10.22	−11.61	36.77	39.99	37.35
SAK_RS04730	Phosphoenolpyruvate-protein phosphotransferase	−8.45	−12.97	−10.77	33.26	35.05	33.91
SAK_RS08555	Asp23/Gls24 family envelope stress response protein	−11.43	−11.72	−11.51	5.40	5.63	5.46
SAK_RS08310	Serine/threonine transporter SstT	−11.05	−11.32	−11.12	27.60	29.63	27.60
SAK_RS03415	DNA-binding protein WhiA	−10.64	−7.93	−10.72	17.68	18.33	17.99
SAK_RS07615	Ribonuclease R	−12.51	−7.61	−10.99	45.71	44.71	44.70
SAK_RS03040	Helix-turn-helix transcriptional regulator	−10.80	−5.70	−10.89	1.91	1.73	1.92
SAK_RS10535	Helix-turn-helix domain-containing protein	−10.84	−6.47	−10.89	14.16	14.27	14.17
SAK_RS04330	DUF3270 domain-containing protein	−12.55	−12.83	−10.30	7.50	7.71	7.52
SAK_RS10205	DUF1292 domain-containing protein	−10.33	−10.28	−12.40	6.02	5.90	6.02
SAK_RS05925	DUF1831 domain-containing protein	−11.42	−6.30	−11.49	13.09	12.33	13.19

### CsbD contributes to the survival of GBS at its later growth period in bile.

**FIG 2:**
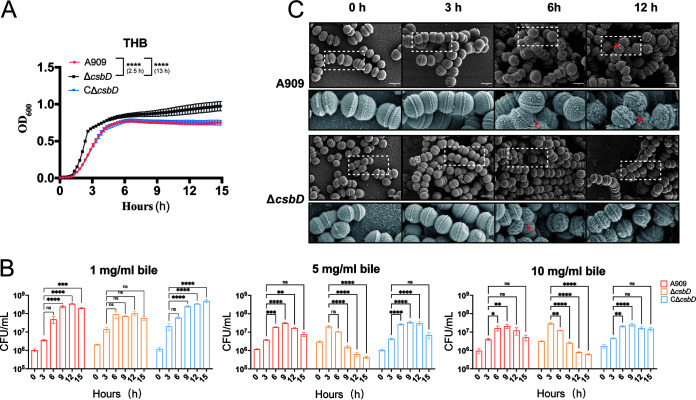
CsbD contributes to the survival of GBS at its later growth period in bile (A) The growth curve of WT, Δ*csbD*, and CΔ*csbD* in THB measured by OD_600_. Data are representative of three independent experiments. Two-way analysis of variance (ANOVA) was used to evaluate the statistical significance of the OD_600_ of WT and Δ*csbD* at different time points (****, *P* < 0.001). (B) The growth curve of WT, Δ*csbD*, and CΔ*csbD* in THB with different concentrations of bile is based on counting the number of CFU. Average CFU/mL standard errors of the means (SEM) for six independent experiments are plotted. Statistical significance relative to the 3 h control was detected at 9 h, 12 h, and 15 h. Two-way ANOVA was used to evaluate statistical significance. ns, *P* > 0.05; *, *P* < 0.05; **, *P* < 0.01; ***, *P* < 0.05; ****, *P* < 0.001. (C) SEM analysis of bile-induced damage of WT and Δ*csbD*. TEM images at 15K. The white dotted box indicates the region is magnified at the bottom. Red arrows indicate the damaged surface present on select strains. The white scale bar represents 1 μm.

Three rounds of A909_Tn_ screening based on 1 mg/mL bile salts included the *csbD* gene, indicating this gene has a strong association with the bile salt tolerant capability, although there is some research indicating that the *csbD* gene was probably a general stress response protein (30, 31). However, its biological function on bile salt resistance has not been well investigated yet. To study the contribution of the *csbD* gene to the survival of the bacteria in bile salts, we constructed a *csbD* mutant (Δ*csbD*) derived from the GBS wild-type (WT) strain A909 and the coordinate complement strain (CΔ*csbD*) as well (Fig. S2A). The deletion of the *csbD* gene slightly increased the bacterial growth rate and elevated the bacterial density at the stationary stage (Fig. 2A). However, the growth advantage was not helpful to Δ*csbD* when cultured in THB with bile salts, especially after culturing for 9 h. As shown in Fig. 2B, the growth curves of WT, Δ*csbD*, and CΔ*csbD* in THB with different concentrations of bile salts were monitored for 15 h by colony-forming units (CFUs) counting. Interestingly, the Δ*csbD* could maintain its bile salt resistance as stable as the WT within the first 3 h even at the 10-mg/mL bile salt concentration, and it still kept the growth advantage overwhelming the WT and complement strain at both 5 mg/mL and 10 mg/mL bile. However, the CFU of the Δ*csbD* started to show a significant decline at 9 h compared to the CFU of Δ*csbD* at 3 h and then kept decreasing along with time, while the growth defect could be restored in the CΔ*csbD* strain, raising the possibility that *csbD* was vitally functional to bile salt resistance in the later growth period of GBS.

Scanning electron microscopy (SEM) analysis was also performed to observe the morphology change of WT GBS and Δ*csbD* under 5 mg/mL bile salts in THB (Fig. 2C). In the control group (0 h) and 3-h growth group, both WT and Δ*csbD* were spheres with a smooth and clear surface, whereas, after 6 h of growth, some of the Δ*csbD* could not maintain a sphere but showed morphologic abnormality, and their surface started to shrink and appear to be rough. This phenotype became more significant and broader after 12 h of growth when GBS lost the *csbD* gene. In contrast, most of the WT strains grew in THB with bile salts and could maintain the normal morphology within a 6-h culture. As the 12-h treatment of 5 mg/mL bile salts, which has detergent activity, put serious pressure on and was even harmful to WT GBS, there were very few WT individuals who showed similar morphologic abnormality as Δ*csbD* at 12 h. Together, these data addressed our hypothesis that the *csbD* gene participated in the bile salt resistance of GBS, especially at the later stage of bacterial growth in bile salts, and the bacterial morphology would be disrupted by bile salts.

### The transcription profile regulated by the *csbD* gene of GBS in bile salts at 12 h.

**FIG 3:**
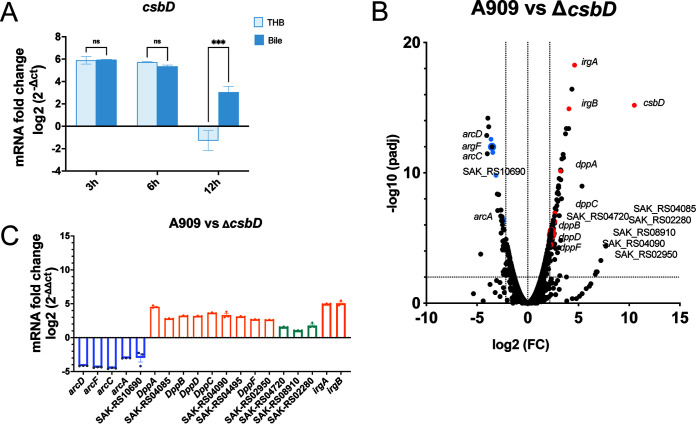
The transcription profile regulated by the *csbD* gene of GBS in bile salts at 12 h. (A) Transcription analysis of *csbD* gene by qRT-PCR in WT cultured in THB with or without 5 mg/mL bile. The data are shown as the means ± SEM from three independent experiments. Two-way ANOVA was used to evaluate statistical significance. ns, *P* > 0.05; ***, *P* < 0.05. (B) RNA-seq analysis by volcano plots showed different expression genes of WT versus Δ*csbD* cultured with 5 mg/mL bile for 12 h. The log_2_ fold change (FC) 2.17 was considered the threshold. The blue points and red points represent downregulated and upregulated known bile tolerance genes, respectively. (C) qRT-PCR analysis of 19 genes to verify the RNA-seq outcomes. The qRT-PCR was performed on three independent RNA samples isolated from WT grown with 5 mg/mL bile. The relative fold changes ± SEM from the log_2_ (2^−ΔΔ^*^CT^*) values are plotted for the 19 genes. The genes with the same color are categorized to the same bile tolerance biological procedure.

According to the growth curve and morphology observed by SEM of Δ*csbD* under bile salt suppression, we believed that the *csbD* was responsible for the bile salt resistance at the later growth stage of GBS. To address this conclusion, we detected the transcription level of the *csbD* gene at different time points under 5-mg/mL bile salt treatment and cultured with THB as control (Fig. 3A). There was no difference in *csbD* gene transcription after encountering bile for 3 or 6 h compared to THB, but the transcript level of *csbD* gene in bile at 12 h was significantly higher than that of THB control group.

The capacity of bile salts to influence gene transcription is well-known in many bacteria (18). To explore whether *csbD* contributes to bile salt resistance by regulating other genes, we performed high-throughput RNA sequencing (RNA-seq) analysis. WT and Δ*csbD* were cultured with 5 mg/mL bile salts for 12 h to identify the transcription profile related to the *csbD* gene in GBS. During growth in the presence of bile salts, there were a total of 279 different transcription genes (DTGs) of WT compared to those in Δ*csbD*, of which 178 genes were upregulated and 101 genes were downregulated. Overall, we used log_2_ fold change of 2.17 as the threshold for significantly different transcription levels to acquire more accurate results. The change in gene transcription was visualized using a volcano plot (Fig. 3B). Eighty-seven genes influenced by *csbD* were upregulated in the presence of 5 mg/mL bile salts. Many DTGs could be categorized to the known bile salts tolerance functions; for example, several genes were dedicated to DNA repair, such as redox NrdH and YbaB/EbfC family nucleoid-associated protein (32, 33). PepSY domain-containing protein and GlsB/YeaQ/YmgE family stress response membrane protein, predicted to be a membrane protein, might consist of a barrier to the influx of bile salts. Twelve genes, ABC transporter (*dppA*, *dppB*, *dppC*, *dppD*, *dppF*, *fetA*, *fetB*, SAK-RS04495, as well as SAK-RS02950) and *IrgA* transporter, involved in transporting substances, were also identified. Furthermore, 33 genes were downregulated in the presence of bile salts. To verify the RNA-seq results, the transcription of 19 genes mentioned above was assessed by quantitative reverse transcription-PCR (qRT-PCR) (Fig. 3C). The results were consistent with those obtained from the RNA-seq analysis, which confirmed the accuracy of the transcriptome changes obtained by RNA-seq in this study.

### CsbD contributes to bile salt resistance by promoting transporter transcription.

**FIG 4:**
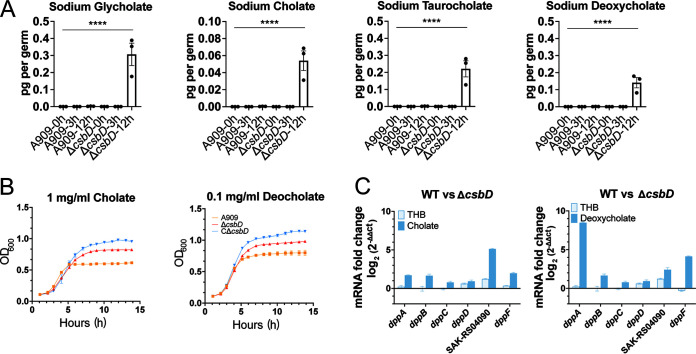
CsbD contributes to bile resistance by promoting transporter transcription. (A) The content of four components within ox bile extract was detected in WT and Δ*csbD* after 0 h, 3 h, and 12 h of culture in THB with bile by HILIC-LC/MS. One-way ANOVA was used to evaluate statistical significance. ****, *P* < 0.001. (B) The growth curve of WT, Δ*csbD*, and CΔ*csbD* in THB with 1 mg/mL cholate or 0.1 mg/mL deoxycholate. (C) Transcription analysis of *dppA*, *dppB*, *dppC*, *dppD*, SAK-RS04090, and *dppE* genes in THB with 1 mg/mL cholate or 0.1 mg/mL deoxycholate by RT-PCR. The data are shown as the means ± SEM from three independent experiments. ****, *P* < 0.001.

The bile salt extrusion by efflux pumps has been proven as an important mechanism that contributes to bacterial bile salt resistance (34). Although the cell wall provides a barrier that reduces bile salts absorption, bile salts can still enter cells by diffusion (35). Therefore, it is necessary to actively efflux to reduce the intracellular concentration of bile salts. Transcriptome analysis showed the gene expression of ABC and IrgAB transporters was upregulated by *csbD* under bile salt conditions. The ABC transporter represents one of the five families associated with multidrug resistance (MDR) that actively excrete harmful substances from the bacterial cytosol (36). Based on that, we hypothesized that *csbD* could contribute to bile salt resistance by promoting the transcription of efflux pump genes. To address the hypothesis, the bile salt content in the WT and the Δ*csbD* grown in THB media with or without bile salts for 12 h was evaluated by HILIC-LC/MS. Previous reviews showed that GBS is abundantly present in the large intestine, where primary bile acids can be converted to secondary bile acids (17, 37). Therefore, four components including primary bile acids and secondary bile acids were detected, respectively, by HILIC-LC/MS, which are also the majority of components of crude ox bile salts extract. The result showed that the sodium salts of taurocholic, glycocholic, deoxycholic, and cholic acids in bacteria grown in 5 mg/mL bile salts THB medium were all accumulated intracellularly. In contrast, their intracellular concentration maintained at a low level in the WT strain under the same cultural environment (Fig. 4A and Fig. S3A). The conjugated bile acids taurocholic and glycocholic are less common in the cecum and rectum. In contrast, unconjugated bile acids accounted for the greatest proportion of all measured bile acids in the large intestine (38). To identify whether *csbD* could be responsible for single component of bile salts in GBS bile resistance, we used sodium deoxycholate and sodium cholate to examine growth curves of WT and Δ*csbD*. The results showed that Δ*csbD* had significant defect in growth compared to the WT at later growth stage (Fig. 4B). Due to the upregulation of transporter genes and low intracellular concentration of bile salts in WT, we anticipated that the capability of bile salt efflux in GBS was enhanced by *csbD*. To test this possibility, we detected the expression profiles of the six genes of ABC transporters randomly selected from the RNA-seq by qRT-PCR in sodium deoxycholate and sodium cholate. As anticipated, all six genes of ABC transporters were upregulated in the Δ*csbD* compared to the WT strain (Fig. 4C). Taken together, the transporter genes regulated by the *csbD* could play a critical role in maintaining resistance against bile salts by reducing the intracellular accumulation of bile salts.

## DISCUSSION

Bile is functional in shaping the composition of the intestinal microbiota. The intestine-colonized bacteria have to resist the antibacterial compounds in bile, which can disrupt bacterial membranes, denature proteins, chelate iron, and calcium and cause oxidative damage to DNA (35). Four main strategies have been summarized, which are widely used by bacteria in bile salt tolerance: (i) exclusion of bile, (ii) extrusion of bile, (iii) repair and defense against damage, and (iv) modulation of virulence (35). The intestinal GBS could be a source of infection that results in serious diseases among neonates, pregnant women, as well as nonpregnant adults (2–4). The bile salt resistance capability of GBS has been known for decades (22); however, the underlying mechanism still needs to be investigated further. For the first time, this work identifies that GBS can minimize the intracellular concentration of bile salts to resist their antibacterial functions. This strategy is relevant to the stress response gene *csbD*.

We first used A909_Tn_ to identify the bile salt resistance genes of GBS. The *csbD* gene identified by TIS-seq was proven to be involved in bile salt resistance in our study. As a general stress response protein, the exact functional role of CsbD is not yet known. In addition to involvement in bile stress response, CsbD has been shown to be involved in phosphate starvation, heat, acidic, oxidative, and salt stresses. It is controlled by several sigma factors, depending on the microorganism: RpoB in Bacillus subtilis, RpoS in Escherichia coli, and RpoT in Pseudomonas putida (30, 39, 40). Based on previous RNA-seq results, *rpoB*, *rpoC*, *rpoD*, *rpoE*, and *rpoZ* transcription is changed in GBS during bile culturing. According to sequence alignment, RpoB in Bacillus subtilis and RpoB in GBS were homologous with 65% sequence identity at the amino acid level. Based on RNA-seq and sequence alignment, it is reasonable to assume that in strain A909 the production of CsbD is regulated by RpoB that encodes the beta subunit of RNA polymerase. The main function of SigB-dependent general stress proteins is to confer nonspecific, focused, and preemptive stress resistance on stressed or starved cells (41). Unlike the *csbD* gene that responded to the bile salt stress signal, several genes related to the biogenesis of the cell wall were increased in the presence of bile salts in our screen, including the *rodA* gene involved in peptidoglycan biosynthesis. The bacterial cell wall maintained by the peptidoglycan layer preserves the integrity of the shape and acts as a barrier against harmful toxins (42). We hypothesized that the deletion of the *rodA* gene damaged the integrity of the bacterial cell wall and made the GBS sensitive to environmental stress. To address this hypothesis, we monitored the growth curve of WT, Δ*rodA*, and the plasmid complement strain CΔ*rodA* in the THB medium with 1 mg/mL, 3 mg/mL, or 5 mg/mL bile salts, respectively (Fig. S4A). The Δ*rodA* became significantly susceptible to bile salt concentration, while the complemented strain may be as resistant as the WT. The growth defect of the WT GBS and *rodA* mutant strains in a range of pH and salt content further proved that defects in peptidoglycan synthesis impair the ability to withstand external stress (Fig. S4B and S4C).

The SEM analysis showed the Δ*csbD* cell surface damage was caused by bile salts, while a similar phenomenon was also found in S. aureus after being exposed to a high concentration of bile salts (43). Once bile salts enter cells, they can cause damage and result in cell death (44). For Gram-positive *Lactobacilli* and S. aureus, when bile salt accumulated in the cell cytoplasm, there is a series of reactions including causing acidification, a loss of proton gradients, and electrical potential across the membrane leading eventually to cell death (43). In our data, the intracellular bile salts accumulated to a higher level compared with WT, especially within 12 h of encounter with bile. Notably, the transcription level of the *csbD* gene at 12 h under the 5-mg/mL bile salt treatment was higher than culture in the absence of bile salts, while this difference was not apparent at the logarithmic phase (3 and 6 h). Our speculation was that the *csbD* gene could mainly participate in the early stage of GBS growth under normal environmental conditions; however, along with the accumulation of bile in GBS, the intensive bile stress-induced *csbD* transcription tended to be vital for bacterial stress adaptability at the stationary phase.

The efflux of bile salts from inside the bacterial cell is energy consuming, and once this process is impeded, the accumulation of harmful substances leads bacteria to death. The Gram-positive bacterial efflux pumps for bile salt extrusion have been identified in S. aureus and E. faecalis (45). Based on the RNA-seq, 12 upregulated genes in WT were involved in transporting substances after culturing in THB with bile salts for 12 h. Of the 12 genes, *dppA*, *dppB*, *dppC*, *dppD*, *dppF*, *fetA*, *fetB*, SAK-RS04495, and SAK-RS02950 all belong to ABC transporters. The ABC transporters belong to the MDR family of efflux pumps using primary active transport to expel compounds and require ATP hydrolysis, not only enabling them to resist the bactericidal effects of bile but also giving them an effective strategy for bacteria to achieve resistance to a multitude of antibiotics (46). The induction of many ABC transport genes expression suggests a possible role of the *csbD* gene in influencing the export of bile salts or bile-derived compounds. As a common bacterial strategy, bile salt transport can counteract bile salt toxicity, and several bile response efflux pump genes have been previously demonstrated in E. coli and L. monocytogenes (47). Moreover, a markedly lower accumulation was detected in our study by HILIC-LC/MS in WT than Δ*csbD* mutant, suggesting that the *csbD* significantly influences bile salt accumulation in GBS during the response to bile salt stress. Ultimately, our research revealed the potential bile salt resistance mechanisms of GBS conferred by the *csbD* gene at bacterial later growth stage under bile salt stress and deepened the understanding of the intestinal colonization strategy of GBS before systemic dissemination.

## MATERIALS AND METHODS

### Bacterial strains, plasmids, and growth conditions.

The bacterial strains and plasmids used in this work are listed in Table S1 and S2. S. agalactiae strains were grown routinely in THB or plated on THB medium with 1.5% (wt/vol) agar at 37°C. For the experiment of comparing the bile salt resistance ability, strains were plated on tryptic soy broth agar medium with or without bile salts at 37°C. Bile salts (Sigma S9875; ox bile extract) were used at indicated concentrations. Sodium deoxycholate (Mackin; S817543) and sodium cholate (Mackin; S817544) were used at 0.1 mg/mL and 1 mg/mL, respectively. All media were filter sterilized with a 0.22-μm filter following the addition of bile salts. Escherichia coli strain DH5α was used as the host for plasmids and was cultured in Luria-Bertani (LB) broth or on LB agar medium. When necessary, antibiotics were used as follows: 100 μg/mL spectinomycin (Spc) or 1,000 μg/mL kanamycin (Km) for S. agalactiae and 50 μg/mL Spc or 50 μg/m Km for E. coli.

### Transposon library construction.

A transposon library (Tn library) was constructed in A909 using pMar4s transposon plasmids by modifying a method used for Tn library construction (48, 49). The temperature-sensitive shuttle plasmid pMar4s contains the fragments of temperature-sensitive replication origin and the Spc-resistance gene from pSET4s plasmid and the mariner-based transposon from pMarA (48). The pMar4s has a temperature-sensitive origin of replication in GBS and will only be propagated at the permissive (cooler) temperature. The pMar4s plasmids were introduced into competent A909 cells by electroporation at 2.35 kV, 200 Ω, and 25 μF using the GenePulser Xcell electroporation system (Bio-Rad, USA). Transformants were resuspended in 5 mL of THB supplemented with 0.2% yeast extract and 30 mM sucrose without antibiotics. After being incubated for 3 h at 28°C to allow cell recovery, the transformed cells were plated onto THB agar with Spc and incubated at 28°C for 12 h to 14 h to ensure that pMar4s replicated as an independent plasmid. More than 100 colonies from each plate were scraped into a single tube (1 per plate), with 24 plates in total. Colonies from each tube were transferred in triplicate, respectively, onto 150-mm (circle) THB + 100-μg/mL Spc plates, 150-mm (circle) THB + 1,000-μg/mL Km plates, and 150-mm (circle) only THB plates with 10 times dilution for 24 h at 37°C. The integration frequency was calculated as the ratio of the number of Spc colonies to the total number of colonies on the THB plate. The integration frequency of each tube less than 0.1% (reverified three times) was selected as the insertion mutant. The diluted selective insertion mutants from each tube were taken to inoculate on THB + 1,000 μg/mL Km at an anticipated concentration of 8 × 10^5^ to 1 × 10^6^ colonies per 245-cm^2^ square plate and 150-mm (circle) THB + 100-μg/mL Spc plates to reverify the integration frequency. After being cultured at 37°C for 12 h to 14 h, the integration frequency of each plate was verified to be less than 0.1%. The final Tn library was constructed by spreading >2.3 × 10^6^ colonies to cover the 10-fold TA dinucleotides site of the genome. Then colonies per plate were scraped, and glycerol was added to the outgrowth “input” sample to 25% stored at −80°C for subsequent Illumina sequencing.

### Screening for the bile resistance gene in GBS with transposon insertion site sequencing (TIS-seq).

An aliquot of the frozen library was serially diluted 1:10 three times and, respectively, plated on THB agar as an input library for 12 h at 37°C and THB agar with Km and 1 mg/mL bile salts as an output library for 12 h at 37°C. There are 1.6 × 10^8^ colonies from an output library to cover the 100-fold TA dinucleotides site of the genome. Three biological replications were used to construct the output library. Bacteria from each replication and input library were collected, and 25 μg of genomic DNA was extracted for TIS-seq using the TaKaRa MiniBEST Bacteria Genomic DNA Extraction kit, following the manufacturer’s instructions. Twenty-five micrograms of genomic DNA was then resuspended in 100 μL to a final concentration of 250 ng/μL and sheared to ~200- to 600-bp fragments by sonication (Covaris, USA) at 50 W peak incident power, 10% duty factor, 200 cycles per burst, and 70-s treatment time. After shearing DNA by sonication, the overhangs were blunted with the Blunt Endase kit (NEB). This was followed by ligation of Illumina adapter (fork truncated NH2 primer and index fork adapter were used to prevent extension of the truncated adapter and limit amplification to Tn-associated sequences) and amplification of genomic DNA transposon (a custom primer, Himmer3outA, was designed for the pMar4s transposon plasmid). Then, we attached Illumina P5 and P7 hybridization sequences and barcodes for multiplexing. The final DNA libraries were gel purified on a 2% agarose gel, and DNA fragments ranging from 300 to 500 bp were extracted. Sequencing reactions were performed on an Illumina MiSeq, and data analysis was conducted as described previously (50). Briefly, the adaptor of raw sequencing data was cut off with cutadaptor V3.4; the trimmed sequences were annotated to GBS A909 genome by Bowtie 1.3; the TA_tally files, which contained TA information of the input or output libraries were generated with python script; and TA_Tally files were analyzed with ARTIST pipeline based on Matlab (51).

### Construction of the mutant and complement strains.

The *rodA* and *csbD* gene deletion mutant strains were generated using homologous recombination, as described previously. The left and right arms of the *rodA* and *csbD* genes were amplified using the primer pairs and cloned into pSET4s (52). All primers are listed in Table S3. The pSET4s plasmid, which is a thermosensitive suicide vector, was used to construct the target gene deletion mutant by carrying fused upstream and downstream arm fragments. The constructed plasmids were verified by sequencing. The recombinant plasmids were introduced into competent A909 cells by electroporation at 2.5 kV, 200 Ω, and 25 μF using the GenePulser Xcell electroporation system (Bio-Rad, USA). Transformants were resuspended in THB supplemented with 0.2% yeast extract and 30 mM sucrose recovery broth and then cultured at 28°C for 4 h. Bacteria were spread on THB agar medium with 100 mg/mL Spc and incubated at 28°C for 12 to 24 h. The transformed bacteria were grown at 37°C in THB plus Spc to generate single-crossover mutants. Double-crossover mutants were generated by repeatedly passaging the single-crossover strains at 28°C on THB without Spc. The gene deletions were verified by PCR and Sanger sequencing.

To construct complementation plasmids, the *rodA* and *csbD* genes were amplified by PCR from A909 genomic DNA and inserted into the pSET2 plasmid (53). The constructed plasmid was amplified in E. coli DH5α before electroporation into Δ*rodA* and Δ*csbD*, respectively. These recombinant vectors were then electroporated into the relative mutant and cultured on THB agar medium with Spc. Then, positive clones were verified by PCR.

### Growth curve analysis.

A single colony from each strain was cultured overnight at 37°C and adjusted to an optical density at 600 nm (OD_600_) = 0.5 with fresh THB medium and then diluted 1:100 in THB with or without stressful stimuli. For comparative analysis of strain resistance to pH, WT, Δ*rodA*, and CΔ*rodA* grown overnight were diluted to an OD_600_ of 0.001 in THB at pH 5.8, 6.8, and 7.8; for salt content, WT, Δ*rodA*, and CΔ*rodA* grown overnight were diluted to an OD_600_ of 0.001 in THB containing 0.2 mM, 0.4 mM NaCl; and for growth characteristics, WT, Δ*csbD*, and CΔ*csbD* grown overnight were diluted to an OD_600_ of 0.001 in THB. For the component of bile, WT and Δ*csbD* grown overnight were diluted to an OD_600_ of 0.001 in THB containing 0.1 mg/mL sodium deoxycholate and 1 mg/mL sodium cholate, respectively. Triplicate cultures were all grown at 225 rpm at 37°C in a 96-well plate for 15 h. Bacterial growth was examined every 15 min by monitoring the OD_600_ using a spectrophotometer (Bio-Rad, USA).

To investigate the bacteria survival in bile salts, overnight S. agalactiae strains were prepared and adjusted to an optical density at 600 nm (OD_600_) of approximately 0.6 with fresh THB medium. Cells were pelleted by centrifugation at 5,000 × *g* for 7 min, washed three times, and later resuspended in phosphate-buffered saline (PBS). Then, 10-μL aliquots of the suspension were inoculated in a THB supplement with different concentrations of bile salts at 37°C. Bacterial growth was assessed by serial dilutions and counting CFU on THB agar plates at each time point.

### Bacterial RNA extraction and RNA-seq.

GBS cultures were grown in THB to exponential phase (OD_600_ = 0.7) and then supplemented with 5 mg/mL bile salts for 3 h, 6 h, and 12 h or supplemented with 1 mg/mL cholate or 0.1 mg/mL deoxycholate for 9 h. Total RNA was isolated by the phenol-chloroform method. After lysis, RNA purification was performed with Total RNA kit I (Omega, USA) using the following the manufacturer’s recommendations. DNA was eliminated by DNase treatment (Turbo DNA-free, Ambion), and RNA purity and integrity were verified by 2% (wt/vol) agarose gel electrophoresis and Experion Automated Electrophoresis System (Bio-Rad Laboratories).

Total RNA was used as input material for the RNA sample preparations, and the insert size of the library was detected by Agilent 2100 bioanalyzer. When the insert size met the expectation, qRT-PCR was used to accurately quantify the effective concentration of the library (the effective concentration of the library is higher than that of 2 nM) to ensure the quality of the library. Then, the libraries were pooled according to the effective concentration and the target amount of data off the machine and sequenced by the Illumina NovaSeq 6000. The image data measured by the high-throughput sequencer were converted into sequence data (reads) by CASAVA base recognition. The reference genome of GBS A909 files was downloaded from the genome website directly. The mapped reads of each sample were assembled by String Tie (v1.3.3b) in a reference-based approach. The feature Counts v1.5.0-p3 was used to count the reads numbers mapped to each gene. Differential expression analysis of two conditions was performed using the edgeR R package (3.24.3). The *P* values were adjusted using the Benjamini and Hochberg method. *P*_adj_ < 0.05 and |log_2_ (fold change)| >1 were set as the threshold for significantly differential expression. The RNA-Seq data were submitted to the NCBI with Bio Project accession number PRJNA943926.

### qRT-PCR.

One microgram of RNA was reverse transcribed into cDNA using Hiscript II QRT Supermix (Vazyme, Nanjing, China). The mRNA levels of target genes were quantified by qRT-PCR using a One Step qRT-PCR SYBR green kit (Vazyme, Nanjing, China). The primers used for the qRT-PCR assay are listed in Table S3 in the supplemental material. For the transcription of the *csbD* gene, each group comprises three biological replicates. For the validation of transcriptome results, each group comprises three technical replicates. Data were normalized to that of a reference housekeeping gene (*recA*) and analyzed by the comparative threshold (ΔΔ*C_T_*) or Δ*C_T_* method (54).

### Scanning electron microscope.

Previous work has shown that the appearance of the outer surface of the bacteria in the presence of bile salts can be analyzed by SEM imagery (43). Briefly, untreated and treated WT and Δ*csbD* cultured with 5 mg/mL bile salts were grown for 3 h, 6 h, and 12 h at 37°C. The samples were removed from the cultures, washed with PBS, and then fixed in 0.1 M PBS (pH 7.4) containing 3% glutaraldehyde fixative overnight at 4°C. The fixed cells were collected via centrifugation at 5,000 × *g* and washed three times with PBS. The fixed bacteria were dehydrated with ethanol (30 to 95%). We mounted the dried specimens on aluminum stubs using conductive carbon cement; the specimens were allowed to dry and were then coated with a gold film. We observed the samples with an SEM at 3 kV and ×15,000 magnification.

### Quantification of intracellular bile salts in GBS with LC/MS.

Samples were prepared as follows: WT and Δ*csbD* cultured in THB with or without 5 mg/mL bile salts for 0 h, 3 h, and 12 h at 37°C were centrifuged at 5,000 × *g* for 7 min. The supernatant was removed, and the bacterial pellets were washed with, and then resuspended in 1 mL sterilized 0.1 M PBS (pH 7.4). We then removed 20 μL of 1 mL total supernatant and transferred it to a fresh 96-well plate. CFU in bacterial solutions at each time point was subsequently determined by serial dilution and colony counting on THB agar plates (Table S4). The content of bile salts was calculated in the bacteria. The remaining bacteria were pelleted by centrifugation and frozen at −4°C for subsequent detection.

An LCMS-8050 triple quadrupole mass spectrometer (Shimadzu, Kyoto, Japan) was used for sample analysis. Chromatographic separation was performed using HSS T3 analytical column (2.1 mm × 100 mm, 1.8 μm) with a flow rate of 0.4 mL/min at 40°C. Ammonium formate in water (10 mM, solvent A) and acetonitrile (solvent B) were employed as the mobile phase. A gradient of 0.2 min 25% B, 2.8 min 25 to 95% B, 1 min 95% B, 0.1 min 95 to 25% B, and 2 min 25% B was used. The optimized mass parameters were set as follows: nebulizing gas flow, 3 L/min; drying gas flow, 15 L/min; interface voltage, 3.5 kV; collision-induced dissociation argon gas pressure, 270 kPa; desolvation line temperature, 250°C; and heat block temperature, 400°C; The mass transition for sodium salts of taurocholic, glycocholic, deoxycholic, and cholic acids was set as *m/z* 464.30 > 74.15 (−), 407.40 > 343.25 (−), 391.40 > 343.40 (−), and 514.30 > 124.05 (−), respectively. Data acquisition and analysis were conducted with LabSolutions LCMS software (version 5.53; Shimadzu).

Sodium salts of glycochol have good linearity in the concentration range of 0.002 to ~1 μg/mL, the fitting equation is *Y* = 1384870*X* + 6121.50, and the regression coefficient *r* value is 0.9912; sodium salts of cholate acids have good linearity in the concentration range of 0.002 to ~2 μg/mL, the fitting equation is *Y* = 1130370*X* − 3907.99, and the regression coefficient *r* value is 0.9918; the linearity of sodium salts of deoxycholate in the concentration range of 0.002 to ~0.2 μg/mL is good, the fitting equation is *Y* = 421830*X* − 777.614, and the regression coefficient *r* value is 0.9972; and sodium salts of taurocholate have good linearity in the concentration range of 0.005 to ~1 μg/mL, the fitting equation is *Y* = 951181*X* − 2746.09, and the regression coefficient *r* value is 0.9903.

The mixed standard working solution was measured according to the analysis conditions, the concentration was taken as the abscissa and the peak area as the ordinate, and the standard curve was made by the external standard method. The peak areas were substituted into the standard curve equation to calculate the concentration of each bile acid in the sample to be tested (Fig. S3B).

### Statistics.

Statistical analyses were performed using GraphPad Prism (version 9.0.1) (GraphPad Software, La Jolla, CA, USA). Two-way ANOVA was performed to analyze the data from the experiments for survival percentage, growth curve, and qRT-PCR. Statistical significance was considered at a *P* value of <0.05.

### Data availability.

The TIS data were submitted to the NCBI with BioProject accession number PRJNA909481. The RNA-Seq data generated from this study were submitted to the NCBI with BioProject accession numbers PRJNA943926.

## References

[1] Ali MM, Asrat D. 2022. Variation of invasive neonatal GBS disease across the regions. Lancet Glob Health 10:e776–e777. doi:10.1016/S2214-109X(22)00182-6. 35490694

[2] Francois Watkins LK, McGee L, Schrag SJ, Beall B, Jain JH, Pondo T, Farley MM, Harrison LH, Zansky SM, Baumbach J, Lynfield R, Snippes Vagnone P, Miller LA, Schaffner W, Thomas AR, Watt JP, Petit S, Langley GE. 2019. Epidemiology of invasive group B Streptococcal infections among nonpregnant adults in the United States, 2008–2016. JAMA Intern Med 179:479–488. doi:10.1001/jamainternmed.2018.7269. 30776079 PMC6450309

[3] Collin SM, Shetty N, Lamagni T. 2020. Invasive group B Streptococcus infections in adults, England, 2015–2016. Emerg Infect Dis 26:1174–1181. doi:10.3201/eid2606.191141. 32441619 PMC7258460

[4] Lamagni TL, Keshishian C, Efstratiou A, Guy R, Henderson KL, Broughton K, Sheridan E. 2013. Emerging trends in the epidemiology of invasive group B Streptococcal disease in England and Wales, 1991–2010. Clin Infect Dis 57:682–688. doi:10.1093/cid/cit337. 23845950

[5] Smith EM, Khan MA, Reingold A, Watt JP. 2015. Group B Streptococcus infections of soft tissue and bone in California adults, 1995–2012. Epidemiol Infect 143:3343–3350. doi:10.1017/S0950268815000606. 26418351 PMC9150969

[6] Trivalle C, Martin E, Martel P, Jacque B, Menard JF, Lemeland JF. 1998. Group B streptococcal bacteraemia in the elderly. J Med Microbiol 47:649–652. doi:10.1099/00222615-47-7-649. 9839570

[7] Back EE, O'Grady EJ, Back JD. 2012. High rates of perinatal group B Streptococcus clindamycin and erythromycin resistance in an upstate New York hospital. Antimicrob Agents Chemother 56:739–742. doi:10.1128/AAC.05794-11. 22143529 PMC3264262

[8] Gygax SE, Schuyler JA, Trama JP, Mordechai E, Adelson ME. 2007. Detection of erythromycin and clindamycin resistance genes in group B Streptococcal clinical isolates and cervicovaginal-rectal swabs. Microb Drug Resist 13:119–123. doi:10.1089/mdr.2007.732. 17650964

[9] Raabe VN, Shane AL. 2019. Group B Streptococcus (Streptococcus agalactiae). Microbiol Spectr 7. doi:10.1128/microbiolspec.GPP3-0007-2018. PMC6432937 30900541

[10] Cubria MB, Vega LA, Shropshire WC, Sanson MA, Shah BJ, Regmi S, Rench M, Baker CJ, Flores AR. 2022. Population genomics reveals distinct temporal association with the emergence of ST1 serotype V group B Streptococcus and macrolide resistance in North America. Antimicrob Agents Chemother 66:e0071421. doi:10.1128/AAC.00714-21. 34633844 PMC8765267

[11] Hansen SM, Uldbjerg N, Kilian M, Sorensen UB. 2004. Dynamics of Streptococcus agalactiae colonization in women during and after pregnancy and in their infants. J Clin Microbiol 42:83–89. doi:10.1128/JCM.42.1.83-89.2004. 14715736 PMC321715

[12] Rajagopal L. 2009. Understanding the regulation of Group B Streptococcal virulence factors. Future Microbiol 4:201–221. doi:10.2217/17460913.4.2.201. 19257847 PMC2691590

[13] Shabayek S, Spellerberg B. 2018. Group B Streptococcal colonization, molecular characteristics, and epidemiology. Front Microbiol 9:437. doi:10.3389/fmicb.2018.00437. 29593684 PMC5861770

[14] Persson K, Bjerre B, Hansson H, Forsgren A. 1981. Several factors influencing the colonization of group B Streptococci–rectum probably the main reservoir. Scand J Infect Dis 13:171–175. doi:10.3109/inf.1981.13.issue-3.03. 6797054

[15] Bianchi-Jassir F, Seale AC, Kohli-Lynch M, Lawn JE, Baker CJ, Bartlett L, Cutland C, Gravett MG, Heath PT, Ip M, Le Doare K, Madhi SA, Saha SK, Schrag S, Sobanjo-Ter Meulen A, Vekemans J, Rubens CE. 2017. Preterm birth associated with group B Streptococcus maternal colonization worldwide: systematic review and meta-analyses. Clin Infect Dis 65:S133–S142. doi:10.1093/cid/cix661. 29117329 PMC5850429

[16] Martins ER, Nascimento do OD, Marques Costa AL, Melo-Cristino J, Ramirez M. 2022. Characteristics of Streptococcus agalactiae colonizing nonpregnant adults support the opportunistic nature of invasive infections. Microbiol Spectr 10:e0108222. doi:10.1128/spectrum.01082-22. 35604173 PMC9241740

[17] Landwehr-Kenzel S, Henneke P. 2014. Interaction of Streptococcus agalactiae and cellular innate immunity in colonization and disease. Front Immunol 5:519. doi:10.3389/fimmu.2014.00519. 25400631 PMC4212683

[18] Begley M, Gahan CG, Hill C. 2005. The interaction between bacteria and bile. FEMS Microbiol Rev 29:625–651. doi:10.1016/j.femsre.2004.09.003. 16102595

[19] Ruiz L, Margolles A, Sanchez B. 2013. Bile resistance mechanisms in Lactobacillus and Bifidobacterium. Front Microbiol 4:396. doi:10.3389/fmicb.2013.00396. 24399996 PMC3872040

[20] Begley M, Sleator RD, Gahan CG, Hill C. 2005. Contribution of three bile-associated loci, bsh, pva, and btlB, to gastrointestinal persistence and bile tolerance of Listeria monocytogenes. Infect Immun 73:894–904. doi:10.1128/IAI.73.2.894-904.2005. 15664931 PMC546953

[21] De Boever P, Wouters R, Verschaeve L, Berckmans P, Schoeters G, Verstraete W. 2000. Protective effect of the bile salt hydrolase-active Lactobacillus reuteri against bile salt cytotoxicity. Appl Microbiol Biotechnol 53:709–714. doi:10.1007/s002530000330. 10919331

[22] Davis GH, Pham AV. 1983. A note on bile tolerance in Streptococcus agalactiae. J Appl Bacteriol 54:127–130. doi:10.1111/j.1365-2672.1983.tb01309.x. 6343329

[23] Goodman AL, McNulty NP, Zhao Y, Leip D, Mitra RD, Lozupone CA, Knight R, Gordon JI. 2009. Identifying genetic determinants needed to establish a human gut symbiont in its habitat. Cell Host Microbe 6:279–289. doi:10.1016/j.chom.2009.08.003. 19748469 PMC2895552

[24] Burcham LR, Le Breton Y, Radin JN, Spencer BL, Deng L, Hiron A, Ransom MR, Mendonca JDC, Belew AT, El-Sayed NM, McIver KS, Kehl-Fie TE, Doran KS. 2020. Identification of zinc-dependent mechanisms used by group B Streptococcus to overcome calprotectin-mediated stress. mBio 11:e02302-20. doi:10.1128/mBio.02302-20. 33173000 PMC7667036

[25] Zhu L, Yerramilli P, Pruitt L, Mishra A, Olsen RJ, Beres SB, Waller AS, Musser JM. 2021. Functional insights into the high-molecular-mass penicillin-binding proteins of Streptococcus agalactiae revealed by gene deletion and transposon mutagenesis analysis. J Bacteriol 203:e0023421. doi:10.1128/JB.00234-21. 34124943 PMC8351624

[26] Percy MG, Grundling A. 2014. Lipoteichoic acid synthesis and function in gram-positive bacteria. Annu Rev Microbiol 68:81–100. doi:10.1146/annurev-micro-091213-112949. 24819367

[27] An H, Douillard FP, Wang G, Zhai Z, Yang J, Song S, Cui J, Ren F, Luo Y, Zhang B, Hao Y. 2014. Integrated transcriptomic and proteomic analysis of the bile stress response in a centenarian-originated probiotic Bifidobacterium longum BBMN68. Mol Cell Proteomics 13:2558–2572. doi:10.1074/mcp.M114.039156. 24965555 PMC4188986

[28] Tong S, Lin Y, Lu S, Wang M, Bogdanov M, Zheng L. 2016. Structural insight into substrate selection and catalysis of lipid phosphate phosphatase PgpB in the cell membrane. J Biol Chem 291:18342–18352. doi:10.1074/jbc.M116.737874. 27405756 PMC5000081

[29] Welsh MA, Schaefer K, Taguchi A, Kahne D, Walker S. 2019. Direction of chain growth and substrate preferences of shape, elongation, division, and sporulation-family peptidoglycan glycosyltransferases. J Am Chem Soc 141:12994–12997. doi:10.1021/jacs.9b06358. 31386359 PMC6738341

[30] Akbar S, Lee SY, Boylan SA, Price CW. 1999. Two genes from Bacillus subtilis under the sole control of the general stress transcription factor sigmaB. Microbiology (Reading) 145:1069–1078. doi:10.1099/13500872-145-5-1069. 10376822

[31] Pragai Z, Harwood CR. 2002. Regulatory interactions between the Pho and sigma(B)-dependent general stress regulons of Bacillus subtilis. Microbiology (Reading) 148:1593–1602. doi:10.1099/00221287-148-5-1593. 11988534

[32] Rabinovitch I, Yanku M, Yeheskel A, Cohen G, Borovok I, Aharonowitz Y. 2010. Staphylococcus aureus NrdH redoxin is a reductant of the class Ib ribonucleotide reductase. J Bacteriol 192:4963–4972. doi:10.1128/JB.00539-10. 20675493 PMC2944516

[33] Pal P, Modi M, Ravichandran S, Yennamalli RM, Priyadarshini R. 2021. DNA-binding properties of YbaB, a putative nucleoid-associated protein from Caulobacter crescentus. Front Microbiol 12:733344. doi:10.3389/fmicb.2021.733344. 34777284 PMC8581549

[34] Sistrunk JR, Nickerson KP, Chanin RB, Rasko DA, Faherty CS. 2016. Survival of the fittest: how bacterial pathogens utilize bile to enhance infection. Clin Microbiol Rev 29:819–836. doi:10.1128/CMR.00031-16. 27464994 PMC5010752

[35] Urdaneta V, Casadesus J. 2017. Interactions between bacteria and bile salts in the gastrointestinal and hepatobiliary tracts. Front Med (Lausanne) 4:163. doi:10.3389/fmed.2017.00163. 29043249 PMC5632352

[36] Reilman E, Mars RA, van Dijl JM, Denham EL. 2014. The multidrug ABC transporter BmrC/BmrD of Bacillus subtilis is regulated via a ribosome-mediated transcriptional attenuation mechanism. Nucleic Acids Res 42:11393–11407. doi:10.1093/nar/gku832. 25217586 PMC4191407

[37] Collins SL, Stine JG, Bisanz JE, Okafor CD, Patterson AD. 2023. Bile acids and the gut microbiota: metabolic interactions and impacts on disease. Nat Rev Microbiol 21:236–247. doi:10.1038/s41579-022-00805-x. 36253479 PMC12536349

[38] Xie G, Zhong W, Li H, Li Q, Qiu Y, Zheng X, Chen H, Zhao X, Zhang S, Zhou Z, Zeisel SH, Jia W. 2013. Alteration of bile acid metabolism in the rat induced by chronic ethanol consumption. FASEB J 27:3583–3593. doi:10.1096/fj.13-231860. 23709616 PMC3752538

[39] Duque E, Rodriguez-Herva JJ, de la Torre J, Dominguez-Cuevas P, Munoz-Rojas J, Ramos JL. 2007. The RpoT regulon of Pseudomonas putida DOT-T1E and its role in stress endurance against solvents. J Bacteriol 189:207–219. doi:10.1128/JB.00950-06. 17071759 PMC1797225

[40] Dong T, Kirchhof MG, Schellhorn HE. 2008. RpoS regulation of gene expression during exponential growth of Escherichia coli K12. Mol Genet Genomics 279:267–277. doi:10.1007/s00438-007-0311-4. 18158608

[41] Hecker M, Pane-Farre J, Volker U. 2007. SigB-dependent general stress response in Bacillus subtilis and related gram-positive bacteria. Annu Rev Microbiol 61:215–236. doi:10.1146/annurev.micro.61.080706.093445. 18035607

[42] Egan AJF, Errington J, Vollmer W. 2020. Regulation of peptidoglycan synthesis and remodelling. Nat Rev Microbiol 18:446–460. doi:10.1038/s41579-020-0366-3. 32424210

[43] Sannasiddappa TH, Lund PA, Clarke SR. 2017. In vitro antibacterial activity of unconjugated and conjugated bile salts on Staphylococcus aureus. Front Microbiol 8:1581. doi:10.3389/fmicb.2017.01581. 28878747 PMC5572772

[44] Prieto AI, Ramos-Morales F, Casadesus J. 2006. Repair of DNA damage induced by bile salts in Salmonella enterica. Genetics 174:575–584. doi:10.1534/genetics.106.060889. 16888329 PMC1602091

[45] Sannasiddappa TH, Hood GA, Hanson KJ, Costabile A, Gibson GR, Clarke SR. 2015. Staphylococcus aureus MnhF mediates cholate efflux and facilitates survival under human colonic conditions. Infect Immun 83:2350–2357. doi:10.1128/IAI.00238-15. 25824834 PMC4432758

[46] Rahman T, Yarnall B, Doyle DA. 2017. Efflux drug transporters at the forefront of antimicrobial resistance. Eur Biophys J 46:647–653. doi:10.1007/s00249-017-1238-2. 28710521 PMC5599465

[47] Alsultan A, Alsallami D. 2022. Efflux-mediated bile resistance in gram-positive pathogens. J Pure Appl Microbiol 16:10–17. doi:10.22207/JPAM.16.1.07.

[48] Liu R, Zhang P, Su Y, Lin H, Zhang H, Yu L, Ma Z, Fan H. 2016. A novel suicide shuttle plasmid for Streptococcus suis serotype 2 and Streptococcus equi ssp. zooepidemicus gene mutation. Sci Rep 6:27133. doi:10.1038/srep27133. 27256117 PMC4891806

[49] Le Breton Y, McIver KS. 2013. Genetic manipulation of Streptococcus pyogenes (the Group A Streptococcus, GAS). Curr Protoc Microbiol 30:9D.3.1–9D.3.29. doi:10.1002/9780471729259.mc09d03s30. PMC3920291 24510894

[50] D’Gama JD, Ma Z, Zhang H, Liu X, Fan H, Morris ERA, Cohen ND, Cywes-Bentley C, Pier GB, Waldor MK. 2019. A conserved streptococcal virulence regulator controls the expression of a distinct class of M-like proteins. mBio 10:e02500-19. doi:10.1128/mBio.02500-19. PMC6805998 31641092

[51] Pritchard JR, Chao MC, Abel S, Davis BM, Baranowski C, Zhang YJ, Rubin EJ, Waldor MK. 2014. ARTIST: high-resolution genome-wide assessment of fitness using transposon-insertion sequencing. PLoS Genet 10:e1004782. doi:10.1371/journal.pgen.1004782. 25375795 PMC4222735

[52] Takamatsu D, Osaki M, Sekizaki T. 2001. Thermosensitive suicide vectors for gene replacement in Streptococcus suis. Plasmid 46:140–148. doi:10.1006/plas.2001.1532. 11591139

[53] Takamatsu D, Osaki M, Sekizaki T. 2001. Construction and characterization of Streptococcus suis-Escherichia coli shuttle cloning vectors. Plasmid 45:101–113. doi:10.1006/plas.2000.1510. 11322824

[54] Livak KJ, Schmittgen TD. 2001. Analysis of relative gene expression data using real-time quantitative PCR and the 2(-Delta Delta C(T)) method. Methods 25:402–408. doi:10.1006/meth.2001.1262. 11846609

