# Supplementary material for: CsbD, a Novel Group B Streptococcal Stress Response Factor That Contributes to Bacterial Resistance against Environmental Bile Salts
Source: J Bacteriol. 2023 May 17;205(6):e00448-22. doi: 10.1128/jb.00448-22 (PMC10294668; doi:10.1128/jb.00448-22)
Supplement: Supplemental file 2 — Fig. S1 to S4 and Tables S1 to S5. Download jb.00448-22-s0002.pdf, PDF file, 2.6 MB [file jb.00448-22-s0002.pdf]

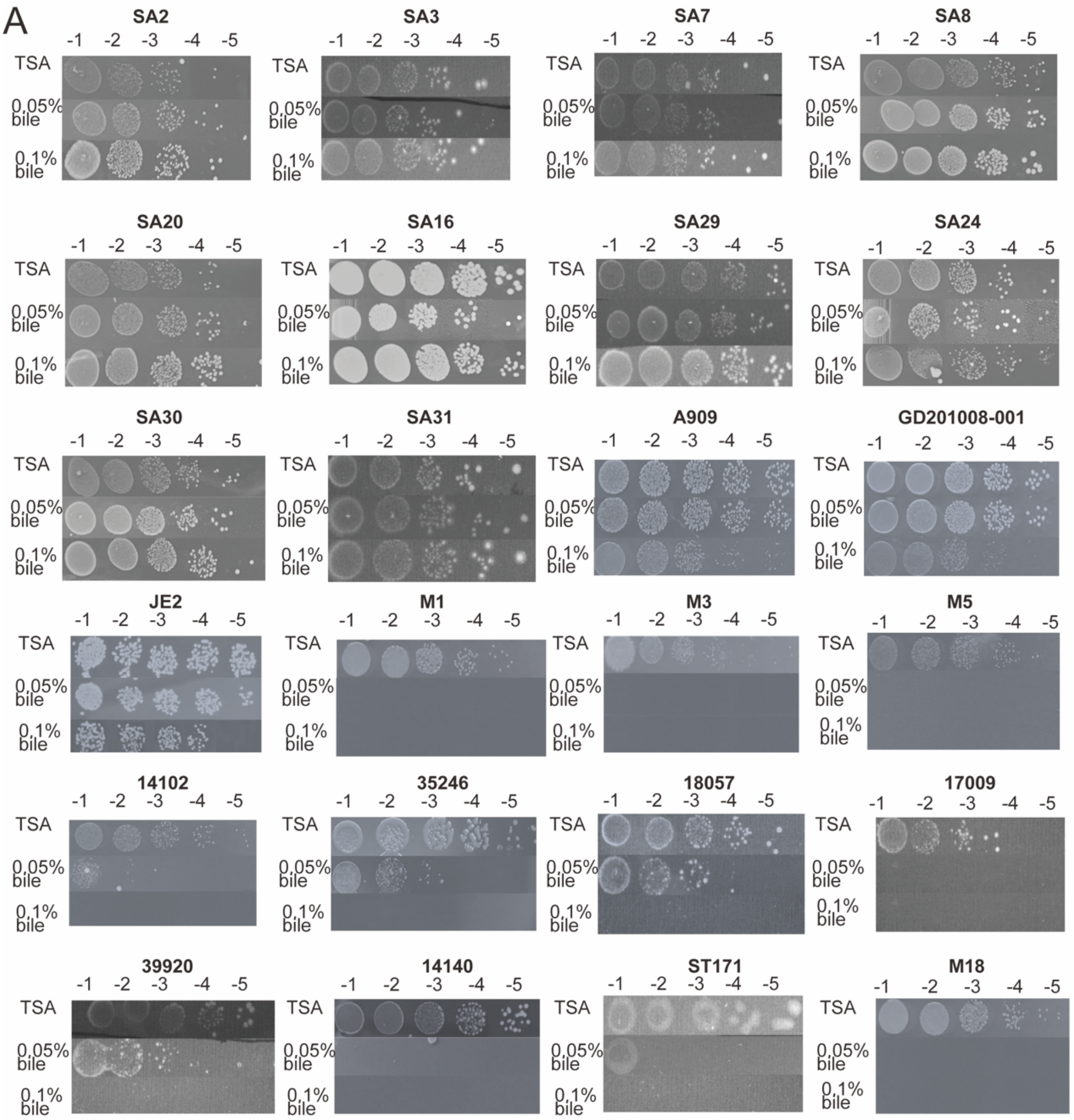

B

HMM Analysis

non-essential for growth genes	3099
essential for optimal growth genes	390
domain essential genes	292

Fig. S1 (A) The bacterial colonies density from 24 strains including GAS, GBS, and GCS at each serial dilution on TSA plates with 0.05 mg/ml or 0.1 mg/ml ox bile extract. (B) HMM analysis genes for GBS fitness in THB.

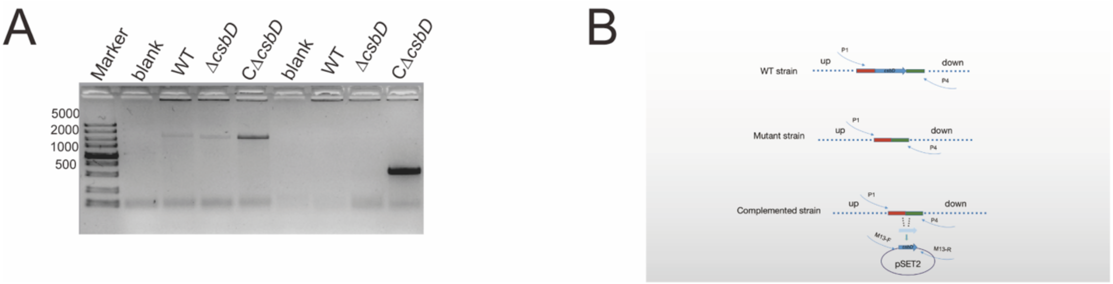

Fig. S2 PCR verification of the WT, Δ*csbD* and CΔ*csbD*. (A) PCR confirmation of Δ*csbD* and CΔ*csbD* using primer pairs *csbD* p1/ p4 and M13 F/R. Shown are strains WT, Δ*csbD* and CΔ*csbD*. The expected PCR products' sizes using primer pairs *csbD* p1/ p4 are 2136 bp for the WT, 1956 bp for the Δ*csbD* and 1956 bp for the CΔ*csbD*. The expected PCR products' sizes using primer pair M13 F/R should be 0 bp, 0 bp and 531 bp. (B) Schematic diagram indicates relative positions of PCR primers and expected PCR products.

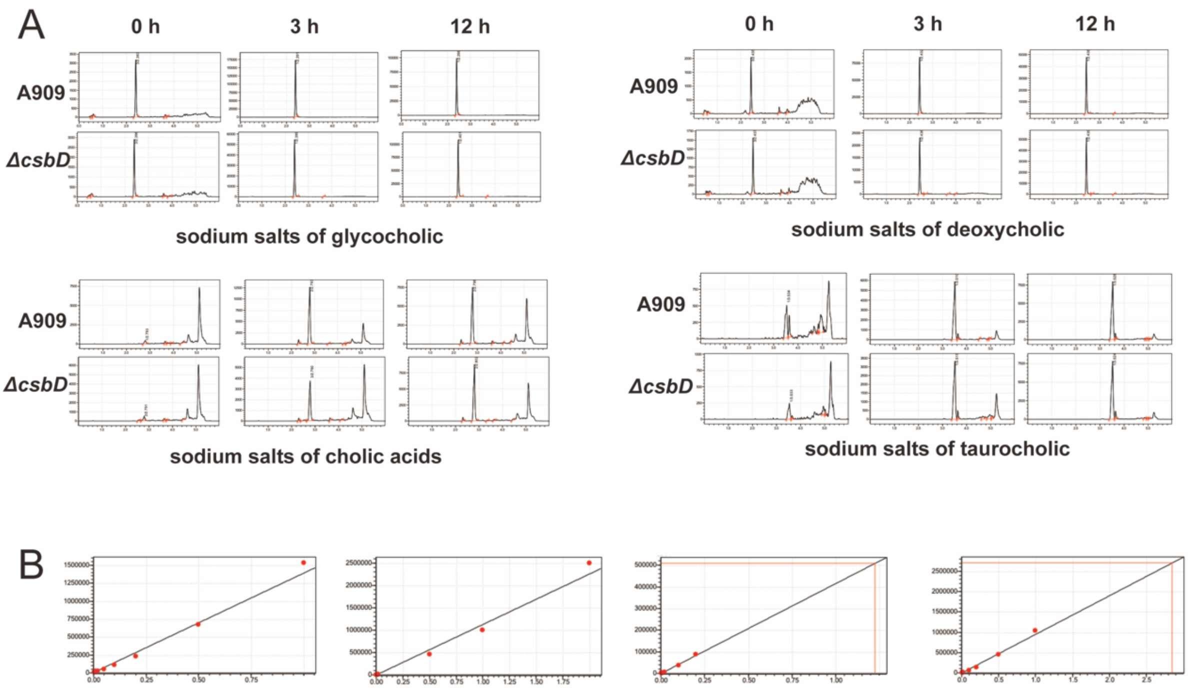

Fig. S3 (A) Chromatograms of sodium salts of taurocholic, glycocholic, deoxycholic. The abscissa represents the retention time of the sample, and the ordinate represents the corresponding intensity of the mass spectrum. Red arrows represent automatically identified peaks. Numbered peaks are representative chromatograms. (B) LC-MS standard curve, from left to right: sodium glycocholate, sodium cholate, sodium deoxycholate, sodium taurocholate. The abscissa represents the different sodium salts of concentration, while the ordinate represents the different sodium salts content (original peak area). Data from three independent experiments.

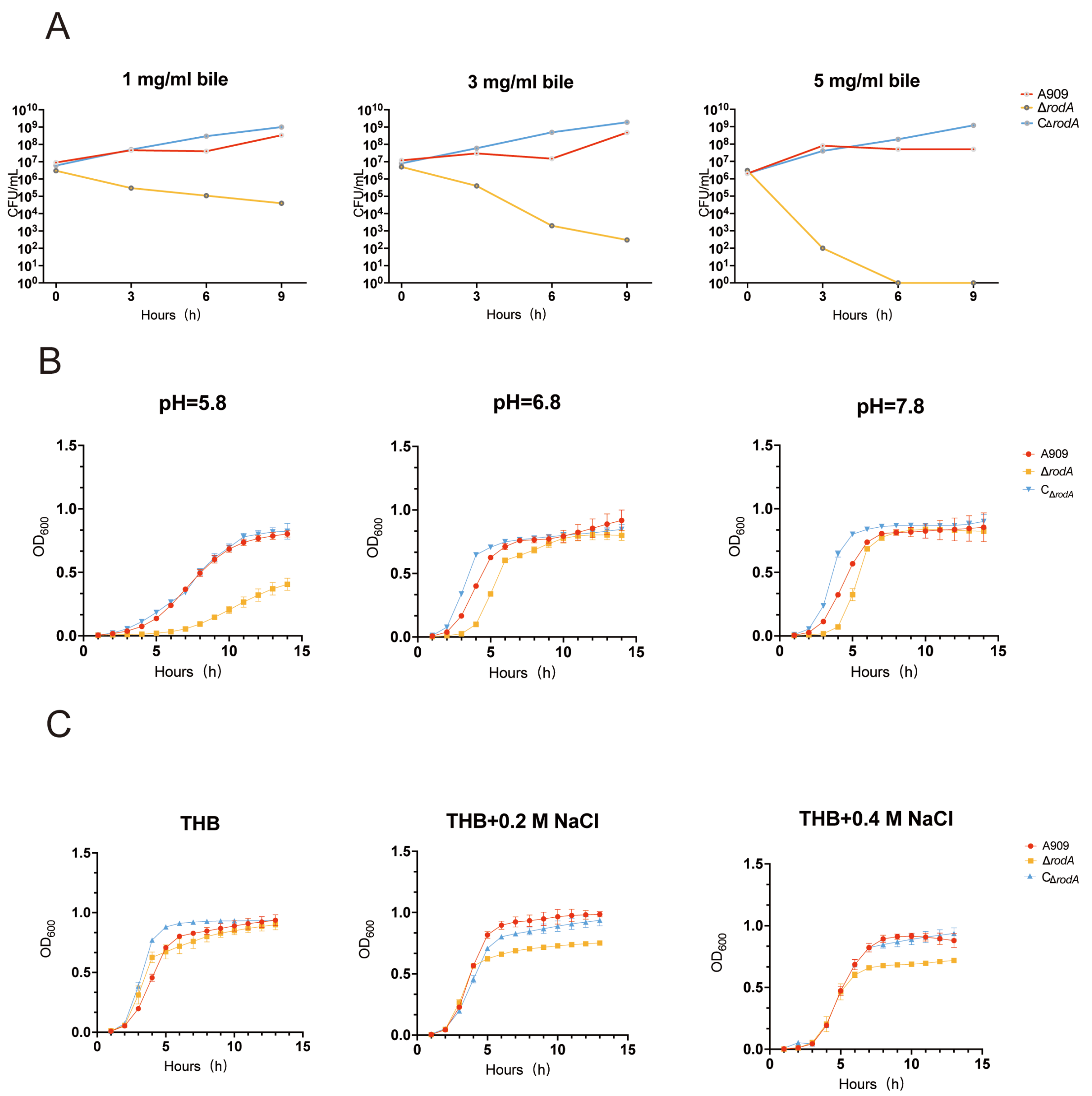

Fig. S4 (A) The growth curve of WT, Δ*rodA*, and CΔ*rodA* in THB medium with 1 mg/ml, 3 mg/ml or 5 mg/ml bile. The Δ*rodA* completely lost its capability to grow in THB with 1 mg/ml, 3 mg/ml or 5 mg/ml bile, while the complemented strain may restore to the WT level. (B) the growth curve of the WT GBS and its derived mutant strains in a range of pH (C) and salt content. The Δ*rodA* showed sensitivity to pH and salt content than the WT and CΔ*rodA*. These results suggested that *rodA* gene contributes to bile salts resistance, pH as well as salt content after *rodA* gene deletion. Data represent the means ± SEM from three independent experiments

Table S1 List of strains used in this research

Strain	Description
Streptococcus agalactiae	
A909	stored in this laboratory
GD201008-001	isolate from tilapia, Guangdong province in China
SA 2	isolate from cow, Tianjing in China
SA 3	isolate from cow, Heibei province in China
SA 7	isolate from cow, Guangxi Zhuang Autonomous Region in China
SA 8	isolate from cow, Nei Monggol Autonomous Region in China
SA 16	isolate from cow, Guangdong province in China
SA 20	isolate from cow, Jiangsu province in China
SA 24	isolate from cow, Henan province in China
SA 29	isolate from cow, Shandong province in China
SA 30	isolate from cow, Heilongjiang province in China
SA 31	isolate from cow, Anhui province in China
Δ*rodA*	In-frame deletion of *rodA* loci in A909
complement *rodA* mutant	Δ*rodA* mutant with plasmid pSET2-rodA
Δ*csbD*	In-frame deletion of *csbD* loci in A909
complement *csbD* mutant	Δ*csbD* mutant with plasmid pSET2-*csbD*
Streptococcus pyogenes	
ATCC 19615	purchased from ATCC
CVCC 593	purchased from CVCC
M3	isolated from human
ATCC BAA-572	purchased from ATCC
Streptococcus equi subspecies *zooepidemicus*	
ST171	stored in this laboratory
14102	isolated from horse
18055	isolated from horse
18057	isolated from horse
17006	isolated from horse
39920	stored in this laboratory
Streptococcus equi subspecies *equi*	
14140	isolated from horse
Escherichia coli	
DH5α	Used for production of recombinant plasmids

Table S2 List of plasmids used in this research

Plasmid	Relevant genotype
pMar4s	Transposon delivery vector; Spc, Kan
pSET4s	temperature sensitive shuttle plasmid; Spc
pSET2	complementation plasmid; Spc
pSET2-*rodA*	*rodA* complementation plasmid; Spc
pSET2-*csbD*	*csbD* complementation plasmid; Spc

Table S3 List of primers used in this research

Primer name	Primer sequence	function
Mutant construction		
rodA-P1	AAACGACGGCCAGTGCAGAGATAGACATATTTCTTATATCCAATAGAAA	
rodA-P2	CCTTTTTTTGAGGACTTCCTTTTTTATTCATGGT	
rodA-P3	AGGAAGTCCTCAAAAAAAGGTTGGAAAGATAATCCA	
rodA-P4	TGACCATGATTACGCCATCTACAGCTGGTCTTCCAG	Construction of *rodA* deletion mutant
csbD-P1	AAACGACGGCCAGTGCTCTTGAGTAATAATCATACTATTGGACTAAT	Construction of *csbD* deletion mutant
csbD-P2	GGTTATTTGACTGCACCTCCTTATTATTTTTATTAAT	
csbD-P3	AGGTGCAGTCAAATAACCGCTCATTTAACGAG	
csbD-P4	TGACCATGATTACGCCATTCTGATGAAAACTAACAGTTATTCG	
Complementation		
rodA-A	GGGGATCCTCTAGAGTCGACCTTGGCAGGGGACATATTAAAAC	Construction of *rodA* complement strain
rodA-B	ATTACGCCAAGCTTGCATGCCACCCTTTATTATATCATATTATTACGATTTTTTATAGC	
csbD-A	CCTCTAGAGTCGACCTTTTAAAACAGGTTATAACACACAAAGTG	Construction of *csbD* complement strain
csbD-B	CCAAGCTTGCATGCCAAAGATAACAAATAACCGCTCG	
M13-F	CCCAGTCACGACGTTGTAAAACG	
M13-R	AGCGGATAACAATTTCACACAGG	
qRT-PCR		
recA q1	ATTCAGGCGCAGTTGATTTAGTT	qRT-PCR for SAK-RS03530
recA q2	TCAATCTCAGCACGAGGAACA	
csbD q1	CTTTGGCAAAATAACCGGTGA	qRT-PCR for SAK-RS03520
csbD q2	TGACAGCATCTTTAGCATCATCT	
dppA-Q1	GGGCAAAGCTTCCTCATGAA	qRT-PCR for SAK-RS01240
dppA-Q2	CATCGTCAGCAGTAACAGGC	
A2-Q1	CAGGAGGAGAAAGCAACGT	qRT-PCR for SAK-RS04085
A2-Q2	GACGACTCTGCTCCTCATCA	
dppB-Q1	GACACGTCACTAACCCCTGT	qRT-PCR for SAK-RS01245

Primer name	Primer sequence	function
dppB-Q2	GACCCGATAAGACCCAGGAG	
dppD-Q1	CCAACTACGCTCCTTTGCAG	qRT-PCR for SAK-RS01255
dppD-Q2	ACTGCTCTCCATTTCCGCTA	
dppC-Q1	GCTTTACGGAACCTTGCTCC	qRT-PCR for SAK-RS01250
dppC-Q2	CCTAAACCTGCAAGCACTCC	
A6-Q1	GCAGCTAAGCGTGGTAAAGG	qRT-PCR for SAK-RS04090
A6-Q2	TTGTCTCCACTTCCTCTTGTTT	
irgA-Q1	CACTACCAATCCCAACGACG	qRT-PCR for *irgA*
irgA-Q2	TCGCTGCTACTGATATTCCTGA	
irgB-Q1	ATTGGAGGGAACACAGCTGT	qRT-PCR for *irgB*
irgB-Q2	CTGCTCCGACTGTATGACCA	
A7-Q1	GAAGCAGCTTACTCGTTGGG	qRT-PCR for SAK-RS04495
A7-Q2	AACTGATTGTGCTCCGTTCC	
DppF-Q1	GTTTATCCAGGGTGCTTGCA	qRT-PCR for SAK-RS01260
DppF-Q2	CCGAGAGTTCACTAGGTCGA	
A9-Q1	CCATAGTTTGAGTGGTGGGC	qRT-PCR for SAK-RS02950
A9-Q2	TCACGAAGATCAGACAACAAGT	
Dna1-Q1	ACAACATTACCAGCTTCAATCAC	qRT-PCR for SAK-RS04720
Dna1-Q2	ACGGCGCTGATTTTGAAGAA	
Dna2-Q1	CAAGAGCTCGTTACTGTGACA	qRT-PCR for SAK-RS08910
Dna2-Q2	ATGGCATTTTCCCCGCAAAT	
Dna3-Q1	GGGCACAGCAGAATACAACC	qRT-PCR for SAK-RS02280
Dna3-Q2	AGCAACATGTTTGGTCTGGC	
Arc1-Q1	TTGTCTTCTCAGGTCGTGCT	qRT-PCR for *arcD*
Arc1-Q2	CTAAGATAGCGCCCCAGTGA	
Arc2-Q1	ACAACGTTGCCAACTCACTT	qRT-PCR for *arcF*
Arc2-Q2	CTCCCATCGATACCCAGACA	
Arc3-Q1	GATGCCAAGAAACAAGCCCA	qRT-PCR for *arcC*
Arc3-Q2	GCCACCTGCGCTAATAACAA	
Arc4-Q1	TGCCATAGATCCGATGCCAA	qRT-PCR for *arcA*
Arc4-Q2	CCGTACTCTGGGTGATGTGT	
Arc5-Q1	ACAGAAGCTCGTTTGACTCG	qRT-PCR for SAK-RS10690
Arc5-Q2	CGAGTGTTTGACAGGATTCCC	
Tn-seq		
Index Fork R	GTGACTGGAGTTCAGACGTGTGCTCTT CCGATCTGGTCGTCGTGGTAT	
Fork truncated NH2	TACCACGACCA	
Himmer 3 out A	CGCAACTGTCCATACTCTG	
Index PCR R	GTGACTGGAGTTCAGACGTGTG	
P5-InvRep-Var1F	AATGATACGGCGACCACCGAGATCTAC ACTCTTTCCCTACACGACGCTCTTCCG ATC TGACTTATCAGCCAACCTGT	

Primer name	Primer sequence	function
P5-InvRep-Var2F	AATGATACGGCGACCACCGAGATCTAC ACTCTTTCCCTACACGACGCTCTTCCG ATC TCGACTTATCAGCCAACCTGT	
P5-InvRep-Var3F	AATGATACGGCGACCACCGAGATCTAC ACTCTTTCCCTACACGACGCTCTTCCG ATC TATGACTTATCAGCCAACCTGT	
P5-InvRep-Var4F	AATGATACGGCGACCACCGAGATCTAC ACTCTTTCCCTACACGACGCTCTTCCG ATC TTGTCGACTTATCAGCCAACCTGT	
P5-InvRep-Var5F	AATGATACGGCGACCACCGAGATCTAC ACTCTTTCCCTACACGACGCTCTTCCG ATC TTCGACGACTTATCAGCCAACCTGT	
P7-AD002-index-R	CAAGCAGAAGACGGCATACGAGAT ACATCG GTGACTGGAGTTCAGACGTGTGCTCTT	
P7-AD004-index-R	CAAGCAGAAGACGGCATACGAGAT TGGTCA GTGACTGGAGTTCAGACGTGTGCTCTT	
P7-AD005-index-R	CAAGCAGAAGACGGCATACGAGAT CACTGT GTGACTGGAGTTCAGACGTGTGCTCTT	
P7-AD007-index-R	CAAGCAGAAGACGGCATACGAGAT CATCTG GTGACTGGAGTTCAGACGTGTGCTCTT	
P7-AD012-index-R	CAAGCAGAAGACGGCATACGAGAT TACAAG GTGACTGGAGTTCAGACGTGTGCTCTT	
P7-AD013-index-R	CAAGCAGAAGACGGCATACGAGAT TTGACT GTGACTGGAGTTCAGACGTGTGCTCTT	
P7-AD014-index-R	CAAGCAGAAGACGGCATACGAGAT GGAACT GTGACTGGAGTTCAGACGTGTGCTCTT	
P7-AD015-index-R	CAAGCAGAAGACGGCATACGAGAT TGACAT GTGACTGGAGTTCAGACGTGTGCTCTT	
P7-AD006-index-R	CAAGCAGAAGACGGCATACGAGAT ATTGGC GTGACTGGAGTTCAGACGTGTGCTCTT	

Table S4 The number of CFU of the detected samples used for HILIC-LC/MS

	**A909 (CFU)**			**Δ*csbD* (CFU)**		
**0 h**	320000000	130000000	170000000	280000000	140000000	110000000
**3 h**	2270000000	2390000000	1030000000	3270000000	2270000000	2970000000
**12 h**	11000000	23000000	15000000	110000	220000	100000

Table S5 The concentration of four components of intracellular bile

	**A909（μg）**			**Δ*csbD*（μg）**		
	0 h	3 h	12 h	0 h	3 h	12 h
Sodium salts of glycocholic	0.00041	0.06148	0.03491	0.00023	0.01935	0.03891
Sodium salts of cholic acids	0.00105	0.00951	0.00616	0.00098	0.00338	0.00686
Sodium salts of deoxycholic	0.00155	0.01333	0.01778	0.00094	0.00835	0.01789
Sodium salts of taurocholic	0.00153	0.04411	0.02591	0.00136	0.01297	0.02804
